# Successful endoscopic treatment of a huge trichobezoar in a 10‐year‐old girl

**DOI:** 10.1002/deo2.357

**Published:** 2024-03-31

**Authors:** Ko Matsuura, Shoji Oura, Kohei Ishibashi, Yoichi Matsumoto, Wataru Ono

**Affiliations:** ^1^ Department of Gastroenterology Kishiwada Tokushukai Hospital Osaka Japan; ^2^ Department of Surgery Kishiwada Tokushukai Hospital Osaka Japan; ^3^ Department of Pediatrics Kishiwada Tokushukai Hospital Osaka Japan

**Keywords:** alligator forceps, dual‐channel multi‐bending scope, FlushKnife, pancreatitis, trichobezoar

## Abstract

A 10‐year‐old girl was admitted to our hospital due to acute pancreatitis. Computed tomography showed an intra‐gastric mass containing multiple small air bubbles. Ultrasound showed a well‐circumscribed large oval mass with a broad acoustic shadow. Endoscopy revealed a huge trichobezoar with many movable hairs, being judged by the cause of acute pancreatitis. Due to the parents’ strong preference not to leave any surgical scars on their daughter, the patient underwent endoscopic treatment. The trichobezoar grasped with a snare was too large to pass through the esophageal‐gastric junction. In addition, the outer layer of the trichobezoar was too hard to be cut with conventional endoscopic devices but was successfully cut with a FlushKnife. The content of the trichobezoar was much softer than its hard surface but needed appropriate counter‐traction to be torn off the tissue. Two alligator forceps via a dual‐channel multi‐bending scope were able to give sufficient counter‐traction to the inner tissue of the trichobezoar, successfully removing the trichobezoar through piece‐by‐piece tearing off. All the endoscopic procedures took seven hours for the complete trichobezoar removal. The total weight of the dissected mass was 180 g. The girl resumed eating on the next day and was discharged on the third day. Physicians should note that a medical team with full endoscopic expertise can remove huge trichobezoars using a FlushKnife, a dual‐channel multi‐bending scope, and two alligator forcepses.

## INTRODUCTION

Bezoars, dissimilar to gallstones and kidney stones, do not cause any symptoms until they become very large and, therefore, have highly been treated with some kind of surgical intervention.^1^ However, advances in endoscopic devices have made it possible to endoscopically remove bezoars of considerable size.^2^ We herein report a huge trichobezoar successfully treated using a FlushKnife,^3^ a dual‐channel multi‐bending scope, and two alligator forcepses.

## CASE REPORT

**FIGURE 1:**
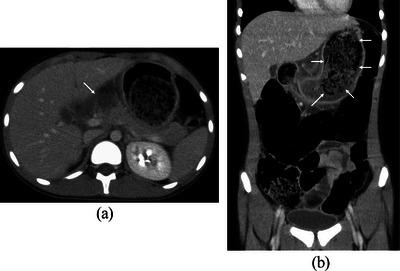
Computed tomography findings. (a) Axial computed tomography showed a non‐enhancing lesion (arrow) in the pancreatic head. (b) Coronal computed tomography showed a large intra‐gastric mass (arrows) with numerous small air bubbles.

**FIGURE 2:**
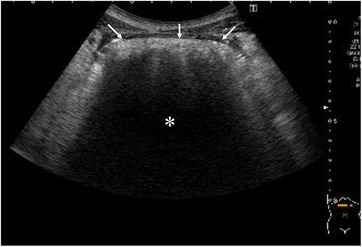
Ultrasonography findings. Ultrasonography showed a large intra‐gastric lesion with smooth borders (arrows) and a broad acoustic shadow (asterisk), causing an impossible evaluation of its inner structure.

**FIGURE 3:**
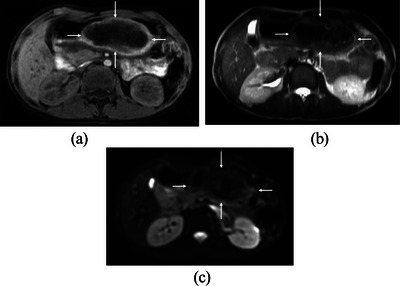
Magnetic resonance imaging. T1‐ (a), T2‐ (b), and diffusion‐weighted (c) images showed a hypo‐intense pattern of the mass.

**FIGURE 4:**
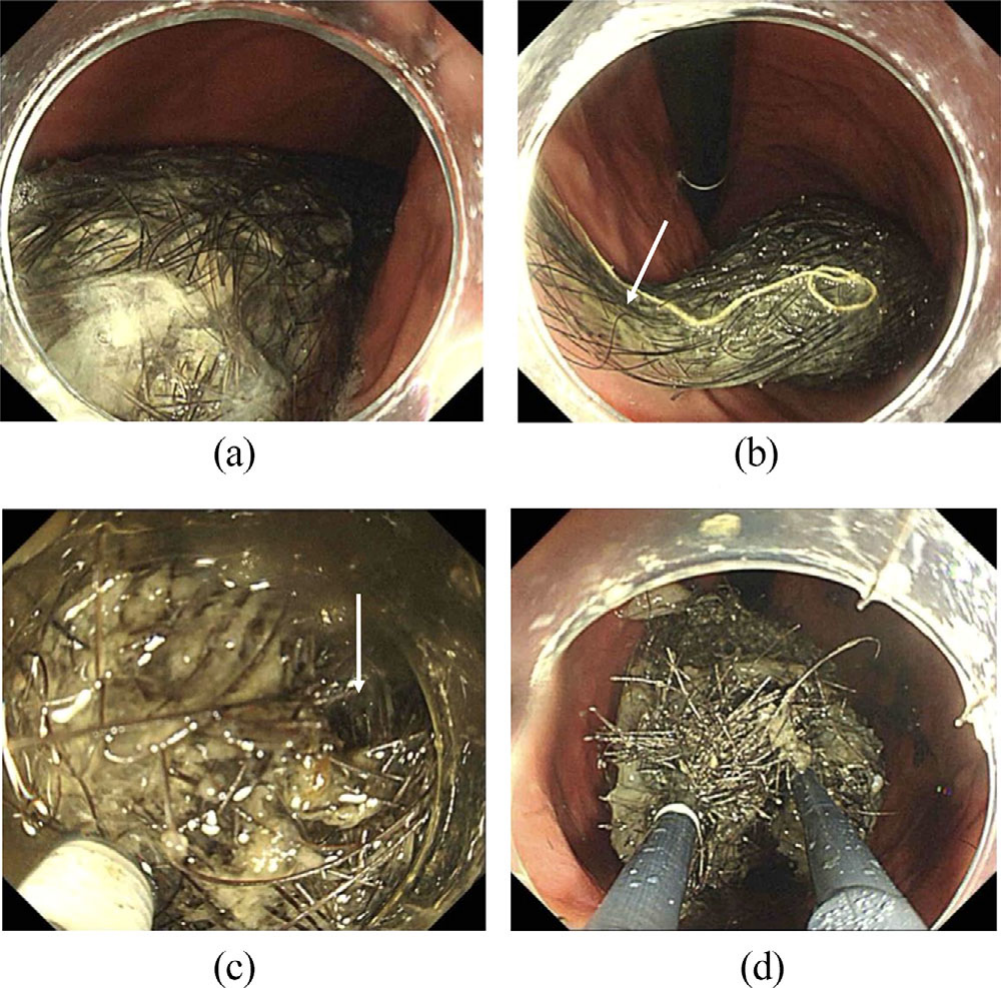
Endoscopic findings and interventional procedures. (a) Since the surface of the large trichobezoar was somewhat transparent, many hairs in the mass were visible. (b) The distal part of the trichobezoar had many movable hairs (arrow). (c) The FlushKnife could efficiently cut the hard surface (arrow) of the trichobezoar. (d) With the two alligator forcepses, the much softer internal content than the mass surface was effectively torn off piece by piece under proper counter‐traction.

A 10‐year‐old girl with an episode of trichophagia developed abdominal pain. Blood tests suggested the abdominal pain caused by acute pancreatitis, that is, a maximum serum amylase level of 813 U/L. Abdominal computed tomography showed interstitial edematous acute pancreatitis and a large intra‐gastric oval mass, 10 cm in size, with numerous small air bubbles (Figure 1). Acute pancreatitis improved after 8 days of fasting and intravenous fluids. Pancreatitis flare‐up, however, was observed 6 weeks from the first onset of acute pancreatitis with a maximum amylase level of 1758 U/L. Ultrasound (US) showed a well‐circumscribed large oval mass with a broad acoustic shadow (Figure 2). Magnetic resonance imaging (MRI) of the mass showed a hypo‐intense pattern on all the T1‐, T2‐, and diffusion‐weighted images (Figures 3a–c). Upper endoscopy showed a large transparent mass containing a lot of hairs (Figure 4a), leading to the diagnosis of trichobezoar. The trichobezoar had many movable hairs from the solid mass (Figure 4b) and was judged as the cause of acute pancreatitis. Due to the parents’ strong request not to leave any surgical scar on their daughter, the patient underwent endoscopic treatment for the trichobezoar. The techniques of endoscopic treatment under general anesthesia were as follows: 1. VIO300D (ERBE) was chosen for the high‐frequency energy device. 2. An overtube, that is, 16 double and short type (TOP), was placed into the esophagus using a GIF‐H290T scope (Olympus, Japan). 3. At first, the trichobezoar could be grasped with a snare but was too large to pass through the esophagogastric junction. The therapeutic strategy of the trichobezoar, therefore, was changed from en block removal to tearing‐off removal. 4. After changing the GIF‐H290T scope to a dual‐channel multi‐bending scope GIF‐2TQ260M (Olympus)^4^ to enable the latter procedures, the trichobezoar was initially attempted to be incised using a Clutch Cutter (FUJIFILM). This attempt unfortunately resulted in only leaving nominal injuries on the mass surface. A shift of the VIO300D energy device setting from ENDO CUT and AUTO CUT to HIGH CUT (Effect 6, 150 W) could neither bring about successful cutting of the hard mass surface. 5. The cutting device, therefore, was changed to a FlushKnife BTS‐B25S (FUJIFILM),^3^ leading to an effective incision of the hard trichobezoar surface (Figure 4c). The inner part of the mass was much softer than the mass surface. However, in order to efficiently remove the trichobezoar, several incisions were also made to the internal tissue with the Flushknife. These incisional procedures generated massive smoke in the operative field, promptly cleared by the sucking out from the other insertion port. Consequently, the mass was torn off piece by piece with two 5‐pronged grasping forceps, that is, so‐called alligator forcepses, under effective counter‐traction (Figure 4D), finally leading to the successful total removal of the huge mass. The time for endoscopic treatment was as long as 7 h. The total weight of the dissected mass was 180g. The patient resumed eating on the next day and was discharged on the 3rd day without any major adverse events. After receiving psychological consultation, the patient has been doing well without further trichophagia and trichobezoar formation for 29 months.

## DISCUSSION

The therapeutic strategy for trichobezoar generally depends on the size of the target lesion. Small trichobezoars can be treated with endoscopic treatment, but small trichobezoars themselves are extremely rarely treated due to the absence of symptoms. Clinical outcomes in this case highly suggest that endoscopic treatment is applicable for the majority of trichobezoars, probably regardless of their sizes.

In performing endoscopic treatment, not only the size of the trichobezoar but also its hardness poses clinically important issues. In this case, the US clearly showed a large intra‐gastric mass with a wide acoustic shadow.^5^ This finding implied the marked difference in the acoustic impedance between the gastric wall and the trichobezoar. Computed tomography findings suggested the acoustic shadow be produced by the numerous air bubbles with a very low acoustic impedance. Therefore, the presence of a wide acoustic shadow itself does not show the hardness of the trichobezoar. In contrast, hypo‐intense patterns all on the T1‐, T2‐, and diffusion‐weighted images also suggested the paucity and/or much less movability of proton in the trichobezoar.^6^ MRI findings, therefore, suggested the trichobezoar either to have some components with little protons such as bone, cartilage, and fibrous components, or to have solid components in which protons can hardly move. These MRI findings highly suggest that the trichobezoar possessed a certain level of hardness, although its constituent components other than hair are unknown.

In this case, acute pancreatitis preceded the diagnosis of trichobezoar. Endoscopic findings highly suggested that many movable hairs hanging down from the mass could have affected the duodenal papilla and caused acute pancreatitis. Detailed mechanisms of developing acute pancreatitis with the trichobezoar remain uncertain due to the lack of endoscopic evaluation around the duodenal papilla in this case. Physicians should note that acute pancreatitis can be a predictor of the presence of trichobezoars.

In the endoscopic treatment of large trichobezoars, Zhao et al. reported the usefulness of the injection of sodium bicarbonate solution into a trichobezoar but needed repeated endoscopic therapy.^7^ It is well known that Coca‐Cola can dissolve persimmon phytobezoars. Matsuoka et al., however, recently reported the usefulness of direct Coca‐Cola injection into a large trichobezoar on endoscopic intervention, resulting in the complete removal of the large trichobezoar in one endoscopic intervention.^8^ Therefore, combination therapy with direct Coca‐Cola injection and our endoscopic intervention techniques can offer a feasible treatment option for the vast majority of large trichobezoars with much less time for endoscopic therapy.

If a thick calcified layer is formed around the trichobezoar, it can be expected that treatment will be more difficult even with our endoscopic techniques. However, given the calcification mechanism in the stomach, it is extremely unlikely that a thick calcified layer is formed around a trichobezoar unless it is present in the gastric diverticulum. In fact, in this case, even minute calcifications were not observed inside or around the trichobezoar on CT.

After the incision failure of the trichobezoar surface with the Clutch Cutter, the FlushKnife could efficiently cut the mass surface. The FlushKnife, however, emits high energy and naturally needs careful handling so as not to injure the gastric wall, especially in the presence of massive smoke that occurs during the endoscopic procedures. In addition to its excellent operability, the dual‐channel multi‐bending scope could allow us to suck out the smoke in the operating field from the other forceps insertion port while one port was in use. In fact, we were able to safely complete the endoscopic procedures without any complications in this case.

In conclusion, in developing a therapeutic strategy for large trichobezoars, open surgery is often considered the first therapeutic option due to the simplicity of its procedures. However, even a small visible surgical scar can have a major impact on a girl's quality of life. Physicians, therefore, should note that endoscopic treatment especially using a FlushKnife, a dual‐channel multi‐bending scope, and two alligator forceps can be a feasible option for large trichobezoar treatment.

## CONFLICT OF INTEREST STATEMENT

None.
